# A systems analysis of the chemosensitivity of breast cancer cells to the polyamine analogue PG-11047

**DOI:** 10.1186/1741-7015-7-77

**Published:** 2009-12-14

**Authors:** Wen-Lin Kuo, Debopriya Das, Safiyyah Ziyad, Sanchita Bhattacharya, William J Gibb, Laura M Heiser, Anguraj Sadanandam, Gerald V Fontenay, Zhi Hu, Nicholas J Wang, Nora Bayani, Heidi S Feiler, Richard M Neve, Andrew J Wyrobek, Paul T Spellman, Laurence J Marton, Joe W Gray

**Affiliations:** 1 Life Sciences Division, Lawrence Berkeley National Laboratory, Berkeley, California, USA; 2 Comprehensive Cancer Center, University of California, San Francisco, California, USA; 3 Progen Pharmaceuticals, Redwood City, California, USA

## Abstract

**Background:**

Polyamines regulate important cellular functions and polyamine dysregulation frequently occurs in cancer. The objective of this study was to use a systems approach to study the relative effects of PG-11047, a polyamine analogue, across breast cancer cells derived from different patients and to identify genetic markers associated with differential cytotoxicity.

**Methods:**

A panel of 48 breast cell lines that mirror many transcriptional and genomic features present in primary human breast tumours were used to study the antiproliferative activity of PG-11047. Sensitive cell lines were further examined for cell cycle distribution and apoptotic response. Cell line responses, quantified by the GI_50 _(dose required for 50% relative growth inhibition) were correlated with the omic profiles of the cell lines to identify markers that predict response and cellular functions associated with drug sensitivity.

**Results:**

The concentrations of PG-11047 needed to inhibit growth of members of the panel of breast cell lines varied over a wide range, with basal-like cell lines being inhibited at lower concentrations than the luminal cell lines. Sensitive cell lines showed a significant decrease in S phase fraction at doses that produced little apoptosis. Correlation of the GI_50 _values with the omic profiles of the cell lines identified genomic, transcriptional and proteomic variables associated with response.

**Conclusions:**

A 13-gene transcriptional marker set was developed as a predictor of response to PG-11047 that warrants clinical evaluation. Analyses of the pathways, networks and genes associated with response to PG-11047 suggest that response may be influenced by interferon signalling and differential inhibition of aspects of motility and epithelial to mesenchymal transition.

See the related commentary by Benes and Settleman: http://www.biomedcentral.com/1741-7015/7/78

## Background

Low molecular weight polycationic polyamines are found in cells in millimolar concentrations and are essential for mammalian cell proliferation, survival and function [1]. Polyamines are associated with nucleic acid metabolism, maintenance of chromatin structure, regulation of specific gene expression, ion channel modulation and membrane stability [1]. The synthesis and catabolism of the polyamines is exquisitely regulated. Polyamines and their biosynthetic enzymes are co-ordinately regulated with growth controls, and polyamine dysregulation frequently occurs in cancer [2]. Thus, targeting this pathway may provide therapeutic advantage in cancer and other hyperproliferative diseases. The polyamine pathway is a downstream target of known oncogenes and the inhibition of polyamine synthesis disrupts the action of these genes [2]. It also appears that the polyamines are critical to the activity of a number of histone deacetylase inhibitors [3]. Thus, the polyamine pathway is a site of therapeutic intervention that is common to, and distal to, a number of validated targets and drugs that interfere with polyamine metabolism and function should have utility both alone and in combination with other agents.

Several approaches to modulating the polyamine pathway have been utilized to date [2]. Targeting the key biosynthetic enzymes has yielded important scientific tools and some clinical success. Difluoromethylornithine, an inhibitor of the first enzyme in the mammalian polyamine biosynthetic pathway, ornithine decarboxylase, is approved for use in trypanosomiasis and has shown promise in the therapy of brain tumours [4]. It has also displayed excellent activity as a chemoprevention for colon polyps, when used in combination with sulindac [5]. However, enzyme inhibitors of the polyamine pathway have generally had limited clinical success to date. A second approach uses dysfunctional synthetic polyamine analogues to competitively inhibit natural polyamine functions. Many of the analogues synthesized to date take advantage of the self-regulatory properties of the natural polyamines and significantly modulate the activity of the biosynthetic and catabolic enzymes, lowering endogenous polyamine levels. Additionally, some of these compounds may function as polyamine mimetics, binding to normal polyamine binding sites in the cell to attenuate normal functions [6].

The compound utilized in this study, PG-11047, is a second generation polyamine analogue of *N*^1^, *N*^12^-bisethylspermine (BESpm) [7]. PG-11047 is a conformationally restricted analogue of BESpm with a cis double bond between the central carbons. It is thought to inhibit proliferation and other cellular functions by competing with natural polyamines. It is active against human cancer cells both *in vitro *[7] and in mouse xenografts [8]. It has shown positive results in the treatment of lymphoma in a phase I human clinical trial as well as in a phase II study for prostate cancer. It has thus far displayed a very benign toxicity profile and a phase I monotherapy trial is nearing completion [2]. It is also in a phase 1b trial in combination with each of seven different approved drugs (Clinical Trial Identifier: NCT00705874). For the combination of PG-11047 with docetaxel or gemcitabine maximally tolerated doses have been reached. Dose escalation of PG-11047 in combination with either bevacizumab, erlotinib, cisplatin, 5-fluorouracil or sunitinib malate is ongoing. With phase II trials emerging as the next step in the evaluation of the efficacy of PG-11047, specific pre-clinical studies are being undertaken to help guide the choice of appropriate clinical subjects. Such preclinical studies include extensive *in vitro *screens in order to provide an insight into the subpopulations of specific cancer types that might be amenable to treatment with PG-11047, while at the same time identifying putative molecular markers for the prediction of sensitivity or resistance to treatment.

Breast cancers develop through multiple genetic and epigenetic changes resulting in a wide range of cancer phenotypes. The resulting breast cancers have been classified, based on their gene expression profiles, into five distinct subtypes that are associated with diverse tumour characteristics and clinical outcome [9,10] and into three groups based on their genomic characteristics [11]. Treatment of a limited number of human breast cancer cell lines in culture with PG-11047 has been reported to inhibit cell growth and/or induce cell death [12,13]. To better delineate the response of breast cancer cells to PG-11047, we studied the antiproliferative activity of this drug in a panel of 48 breast cell lines (including six non-malignant breast cell lines) that mirror many transcriptional and genomic features present in primary human breast tumours [14]. The initial characterization of this panel shows that the cell lines are comprised of three subtypes designated basal A, basal B and luminal. The basal A subtype corresponds most closely to tumours designated as basal-like [15,16] by expression profiling while the basal B subtype corresponds most closely to tumours designated as claudin-low [17] (and Spellman *et al*., personal communication). The basal A and basal B subtypes will be referred to as basal and claudin-low, respectively, in the following text. Our studies show that basal subtype breast cancer cell lines are most sensitive to treatment with PG-11047 based on the dose required to inhibit 50% relative growth (GI_50_). Correlations between cell line responses quantified by the GI_50 _and the transcriptional and genomic characteristics of these cell lines identified a set of 250 genes that are associated with response. A signature comprised of 13 of these genes is proposed as a predictor of response to PG-11047 treatment in breast tumours.

## Methods

### Breast cancer cell lines

Breast cancer cell lines were obtained from the American Type Culture Collection and from collections developed in the laboratories of Drs Steve Ethier (Karmanos Cancer Center, MI, USA) and Adi Gazdar (UT Southwestern Medical Center, TX, USA). Forty of the cell lines were characterized in great detail by Neve *et al*. [14], eight were acquired recently and characterized in similar fashion for subtype classification (Spellman *et al*., personal communication). The recurrent genome copy number abnormalities in the collection of cell lines was similar to those in primary tumours, while hierarchical analysis of the transcriptional features of the cell lines defined three clusters designated luminal, basal and claudin-low [14].

### Cell growth inhibition assay and data analysis

Cells were plated at proper density (from 1000 to 15,000 cells/100 μl/well, depending on the cell line) in 96-well plates, so that they remained in logarithmic growth at the time of assay. The cells were allowed to attach overnight before being exposed to the polyamine analogue PG-11047 for 72 h. PG-11047 (*N*^1^, *N*^12^bis(ethyl)-6,7-dehydrospermine tetrahydrochloride, previously known as SL-11047 and CGC-11047) was obtained from Progen Pharmaceuticals (CA, USA) and a stock solution of 100 mM was prepared in sterile water. For the dose response study, a set of nine doses (final concentration from 5 mM to 13 nM) in 1:5 serial dilution were added in triplicate wells. Adenosine triphosphate content was measured as an estimate of relative cell number using the CellTiter-Glo (CTG) Luminescent Cell Viability Assay (Promega, WI, USA), with slight modification form manufacturer's protocol, at day 0 (time when drug was added) and day 3 of drug exposure. Briefly, the CTG reagent was diluted with phosphate-buffered saline (PBS; 1:1, volume: volume) and the culture media was removed from the 96-well plate prior to adding 50 μl per well of the diluted CTG reagent. Luminescence from the assay was recorded using BIO-TEK FLx800.

Metrics describing the drug effects were calculated according to the methods described by the NCI/NIH DTP Human Tumor Cell Line Screen [18] and by Monks *et al*. [19]. The % growth curve is calculated as ((T-T_0_)/(C- T_0_)) ×100, where T_0 _is the CTG luminescence at day 0, C is the untreated control CTG luminescence at day 3 and T is the CTG luminescence at the test concentration. The dose response curve was fitted with GraphPad Prism4 software (GraphPad Software, Inc, CA, USA). The GI_50 _and TGI (total growth inhibition) values were determined as the drug concentrations that caused 50% and 0% relative growth at 72 h drug exposure, respectively.

### Bromodeoxyuridine (BrdUrd) labelling and cell cycle analyses

The fractions of cells in the G1-S and G2 M phases of the cell cycle were estimated from measurements of BrdUrd-DNA distributions [20]. For this, cells were set up the same as in the cell growth inhibition assay and treated with PG-11047 at three different doses of 0.3, 10 or 300 μM for 48 h and 72 h. A positive control of 10 nM docetaxel treatment was included in all experiments. Cells were pulse labelled with a final BrdUrd concentration of 10 μM for 30 min, then fixed with 70% ethanol overnight in the 96-well plate. Fixed cells were denatured with 2N hydrochloric acid for 30 min and then incubated with primary anti-BrdUrd antibody (BD Biosciences, CA, USA; 1:100 dilutions), followed by staining with a secondary antibody (1:500 dilution) labelled with Alexa-488 and counterstained with Hoechst 33342 (1:2000 dilution). The cells were then placed in PBS and Hoechst 33342 and Alexa-488 images were acquired for cells in each well using an ArrayScan imaging system (Cellomics Inc, PA, USA). The Hoechst images were segmented to localize nuclei and Hoechst fluorescence was measured for each nucleus as an estimate of relative DNA content. Alexa-488 fluorescence was measured for each segmented nucleus as an estimate of incorporated BrdUrd. Several thousand cells were measured for each well and the Hoechst 33342 and Alexa-488 results were combined to produce a bivariate BrdUrd-DNA distribution similar to that produced using flow cytometry [20]. The bivariate distributions for each well were analysed to determine the fractions of cells in the G1, S and G2 M phases of the cell cycle using FlowJo software (Tree Star, Inc, OR, USA). Flow cytometric BrdUrd-DNA distributions measured for several replicate experiments produced cell cycle fractions similar to those obtained using the imaging approach (data not shown).

### Apoptosis assay

The extent of PG-11047-induced apoptosis was determined using the Caspase-Glo 3/7 assay kit from Promega (WI, USA). A positive control of 10 nM docetaxel treatment was included in all experiments. After incubation of cells with PG-11047 at the specified concentrations for 48 h or 72 h, 50 μl Caspase-Glo reagent was added to wells. After an incubation time of 1 h, luminescence was measured using a BIO-TEK FLx800 luminometer. Parallel experiments were set up to determine the relative number of viable cells using the CTG assay, as described above, in order to normalize the caspase activity by relative cell density.

### Statistical analysis of molecular markers associated with response

The PG-11047 GI_50 _levels for all cell lines were correlated with the molecular features measured for each cell lines (microarray profiles for RNA expression and genome copy number and Western blot based protein profiles) as reported by Neve *et al*. [14]. We used a statistical approach based on adaptive linear splines in order to identify the molecular correlates of response to PG-11047. These are variants of the linear splines method described previously [21]. Briefly, we used a non-parametric regression method to model non-linear relationships between molecular correlates and response. The goodness of fit was assessed by evaluating a *P*-value corresponding to the *F*-statistic for the fit [21]. *P*-values were corrected for multiple hypotheses testing using the false discovery rate method [22]. This process identified 250 genes whose transcripts were associated with response. From these, a 13 gene set was developed in order to predict a quantitative response among the cell lines using Monte Carlo cross-validation (MCCV) [23]. The complete panel of cell lines was used for this purpose. In MCCV, the samples (cell lines) are randomly partitioned into training sets and test sets. The marker genes found to be significant in the training set are then evaluated for their predictive accuracy in the test set. This random partitioning process is iterated multiple times. The 13 genes were consistently found to be significant across the iterations. More details are described in [23] (also, Das *et al*, in preparation). The final model was determined via leave-one-out cross-validation. Ingenuity Pathway Analysis (IPA 5.0; Ingenuity Software, Inc, CA, USA) knowledgebase molecular interactome was applied to the 250 predictor genes to identify the networks of genes, generated algorithmically based on their connectivity. Network genes were further analysed for significant pathways associations.

## Results

### Effect of polyamine analogue PG-11047 on breast cancer cell lines

**Figure 1:**
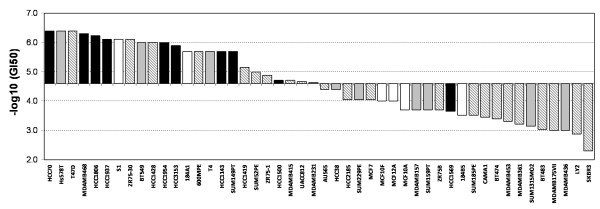
**Growth inhibition (GI_50_) sensitivity profile of the breast cell lines to PG-11047**. Cell lines are arranged from sensitive (left) to resistant (right) based on median centred GI_50 _value. Solid bar, basal; gray bar, claudin low; striped bar, luminal subtype; white bar, non-malignant breast cells.

Forty-two breast cancer cell lines representing luminal, basal and claudin-low breast cancer subtypes and six non-malignant breast cell lines were treated with PG-11047 in doses ranging from 13 nM to 5 mM for 72 h. The GI_50 _dose was calculated for each of the cell lines and ranged from 0.4 μM to 5 mM (Additional File 1) with a median GI_50 _at 31.5 μM. The distribution of GI_50 _values for the cell lines arranged from most sensitive to most resistant is shown in Figure 1 along with subtype classification. These data show that the basal and claudin-low subtypes were inhibited at the lowest levels of PG-11047 (*P *< 0.01; Mann-Whitney test). The TGI (dose required to inhibit 100 percent of the cell growth from T_0_) showed similar subtype specificity (Additional File 1).

### Predictive markers for PG-11047 response

We correlated the GI_50 _values for the cell lines in the panel with their pretreatment genomic, transcriptional and proteomic profiles in order to identify molecular factors associated with cellular response to PG-11047. Additional Files 2, 3 and 4 list mRNA, DNA and protein features that were significantly associated with response. The molecular features associated with response or resistance when present at elevated levels are listed as response predictors.

**Figure 2:**
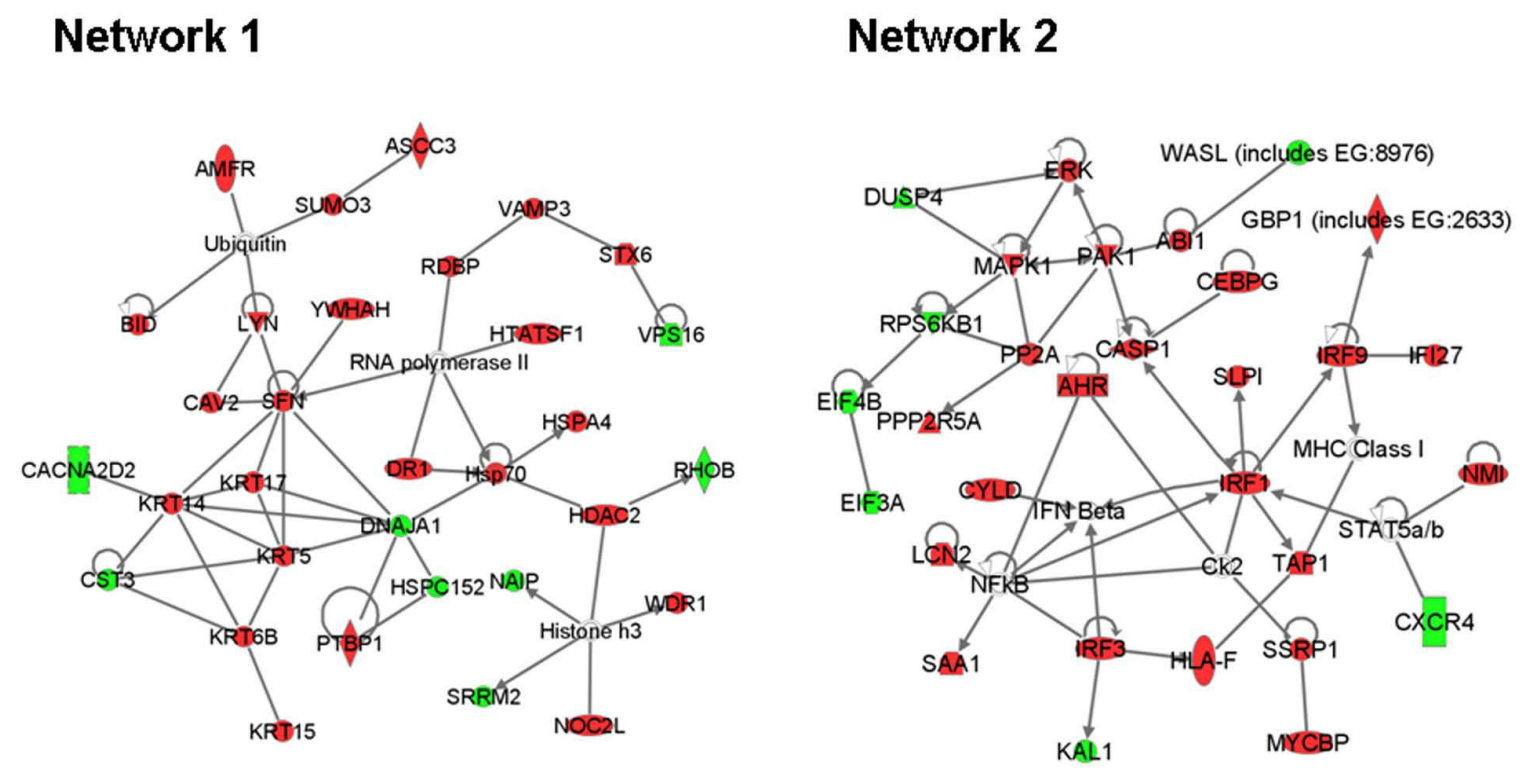
**Network analysis of the top 250 predictor genes**. Ingenuity Pathway Analysis was applied to the top 250 predictor genes to identify gene networks based on connectivity (red: predictor of sensitivity, green: predictor of resistance). The top two networks are shown here. The network scores for networks 1 and 2 are 54 and 49, respectively. Nodes are displayed using shapes that represent the functional class of the gene product. Edges are displayed with labels that describe the nature of the relationship between the nodes. All edges are supported by at least 1 reference from the literature, or from canonical information stored in the Ingenuity Pathways Knowledge Base.

IPA of 250 mRNA transcripts significantly associated with response showed several pathways as significantly associated with response (Table 1). The interferon signalling pathway was most significantly involved in PG-11047 response, with four of the genes identified in the top 250 genes out of a total of 29 genes identified in the pathway. Network connectivity analysis suggested interactions among the 250 genes most significantly associated with response. The most significant two networks are shown in Figure 2. Network 1 involves basal cytokeratins, ubiquitin/proteasome processes, RNA processing and histone deacetylase. Network 2 involves interferon signalling.

**Table 1:** Significant pathways from top 250 predictor genes associated with response to PG-11047 treatment using Ingenuity Pathway analysis.

Pathway Name	P-Value	Ratio	Component genes in the pathway
Interferon Signaling	0.001	[4/29]	IRF1, IRF9, TAP1, IFNGR1

Ephrin Receptor Signaling	0.011	[8/193]	**CXCR4**, ABI1, RAP1A, MAPK1, **WASL**, MAP4K4, GNAI3, PAK1

Role of BRCA1 in DNA Damage Response	0.013	[4/52]	RPA1, RFC3, MSH2, E2F3

Nicotinate and Nicotinamide Metabolism	0.019	[5/129]	NNMT, **ENPP2**, MAPK1, **ENPP1**, PAK1

Fcy Receptor -mediatedPhagocytosis in Macrophages andMonocytes	0.028	[5/104]	**RPS6KB1**, LYN, MAPK1, VAMP3, PAK1

Riboflavin Metabolism	0.032	[2/49]	**ENPP2, ENPP1**

Endoplasmic Reticulum Stress Pathway	0.041	[2/18]	**XBP1**, TAOK3

Pantothenateand CoA Biosynthesis	0.046	[2/63]	**ENPP2, ENPP1**

CXCR4 Signaling	0.052	[6/164]	**CXCR4, RHOB**, LYN, MAPK1, GNAI3, PAK1

PI3K/AKT Signaling	0.055	[5/135]	**RPS6KB1**, SFN, MAPK1, YWHAH, PPP2R5A

Integrin Signaling	0.056	[7/198]	**RHOB**, RAP1A, MAPK1, TSPAN6, **WASL**, ARF4, PAK1

Ratio indicates the number of genes in the top 250 predictors that belong to the pathway indicated over the number of genes known of the pathway. Genes in bold were associated with resistance when transcribed at high levels. Genes in normal fonts were associated with sensitivity when transcribed at high levels.

Further analysis of the 250 mRNA transcript levels associated with response in the cell lines yielded a 13 gene signature as possible clinical predictor of response to PG-11047 treatment (Table 2). Higher levels of expression of WASL, CST3, DEAF1 and ACSL3 were associated with resistance to PG-11047 while high expression levels of GCLM, LAMA3, SSRP1, ACYP1, CYLD, PRPF18, AMFR, PPP1R2 and LOH11CR2A were associated with increased sensitivity. We evaluated the performance of the 13 gene signature in predicting sensitivity of the cell-lines to PG-11047. The average correlation between predicted and measured sensitivities was 0.93. Here, multiple random samplings of training and test sets were performed. The average reflects the performance of the predictive model across these iterations in the test sets. The genes in this signature are involved in cell motility, response to stress and cellular metabolic processes, based on the known functions of the genes.

**Table 2:** Gene list predicting sensitivity (S) or resistance (R) to PG-11047 treatment.

Gene symbol	*P*-value	Predict	Chromosome location	Gene name	Function	References
WASL	2.5E-05	R	chr7q31.3	Wiskott-Aldrich syndrome-like	Key regulator of cell migration and actin polymerization by interaction with Ap2/3 complex	[32,47]

GCLM	4.7E-05	S	chr1p22.1	Glutamate-cysteine ligase, modifier subunit	Regulatory subunit of glutamate-cystein ligase, a rate-limiting enzyme catalyzing the first step of GSK biosynthesis from cysteine	[39]

CST3	6.3E-05	R	chr20p11.21	Cystatin C (amyloid angiopathy and cerebral haemorrhage)	Potent inhibitor of lysosomal proteinases. TGFβ receptor antagonist, inhibit epithelial-mesenchymal transition and morphological transformation	[30]

LAMA3	6.7E-05	S	chr18q11.2	Laminin, alpha 3	Component of laminin 5, the major extracellular metrix protein produced in the basement membrane of the mammary epithelial cells. Induces motility, invasion and epithelial-mesenchymal transition	[35,48]

SSRP1	1.2E-04	S	chr11q12	Structure specific recognition protein 1	Histone chaperone. Forms a heterodimer with Spt16 and is a component of the chromatin transcript regulation factor, FACT	[40,42]

ACYP1	1.2E-04	S	chr14q24.3	Acylphosphatase 1, erythrocyte (common) type	Catalyze the hydroolysis of acylphosphates	[49]

CYLD	2.9E-04	S	chr16q12.1	Cylindromatosis (turban tumour syndrome)	A deubiquinating enzyme that negatively regulate the activity of NFkB and JNK. Regulate mitotic entry	[43,45]

PRPF18	3.0E-04	S	chr10p13	PRP18 pre-mRNA processing factor 18 homolog (*Saccharomyces cerevisiae*)	Participate in pre-mRNA splicing, prepare mRNA for translation	[41]

AMFR	3.1E-04	S	chr16q21	Autocrine motility factor receptor	Receptor for AMF, a tumour motility-stimulating protein secreted by tumour cells. Stimulation of AMFR by AMF alters cellular adhesion, proliferation, motility and apoptosis	[33,36]

DEAF1	7.9E-04	R	chr11p15.5	Deformed epidermal autoregulatory factor 1 (*Drosophila*)	A sequence-specific DNA-binding protein required for development. May be a general regulator of gene transcription/expression. DEAF1 (also called 'suppressin') appear to be mutated in tumours and cell lines	[50,51]

PPP1R2	1.5E-03	S	chr3q29	Protein phosphatase 1, regulatory (inhibitor) subunit 2	Inhibitor of protein phosphatase 1. Phosphorylation of PPP1R2 by glycogen synthase kinase-3 (GSK3) activates the enzyme. PPP1R2 (INH2) regulates PP1 complexed with NEK2 to induce centrosome separation	[44]

LOH11CR2A	1.7E-03	S	chr11q23	Loss of heterozygosity, 11, chromosomal region 2, gene A	Frequent loss of heterozygosity at 11q23 region found in many tumour types	[46]

ACSL3	1.8E-03	R	chr2q34-q35	Acyl-CoA synthetase long-chain family member 3	Essential for fatty acid metabolism, providing activated intermediates for complex lipid synthesis, protein modification, and beta oxidation	[52]

Correlative analysis of GI_50 _sensitivity of the cell line panel with genomic copy number aberration (array comparative genomic hybridization data) and protein level (Western data) identified additional markers significantly associated with response. These are listed in Additional Files 3 and 4. Several of the 13 gene signature transcriptional markers were found in regions of genomic abnormality associated with response. Examples include: genomic copy gains of the chromosomal regions near AMFR (16q21), SSRP1 (11q12) and LOH11CR2A (11q23) and copy number loss of CST3 (20p11.2) that predict sensitivity to PG-11047 treatment (Additional File 3). These observations are consistent with the predictors developed with the mRNA expression data and suggest that genomic aberrations may drive the changes in gene expression that determine response to PG-11047. Interestingly, the status of AKT was among the protein levels significantly associated with response (Additional file 4), with high phospho-AKT associated with sensitivity and high AKT associated with resistance. Not surprisingly, high level expression of the basal subtype cytokeratins, CK5 and CK6, were associated with sensitivity while high level expression of the luminal cytokeratin, CK18 was associated with resistance.

### PG-11047 mediated cell cycle changes

**Figure 3:**
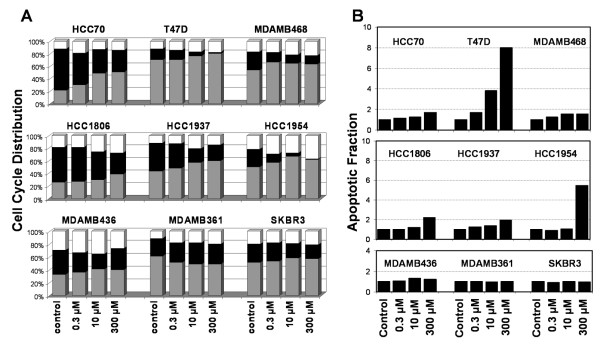
**Cell cycle distribution and apoptotic fraction of PG-11047 treated breast cancer cell lines**. Six sensitive (top two rows) and three resistant (bottom row) cell lines were tested with PG-11047 for 72 h. (A) Fractions of cells estimated to be in the G1 (gray bar), S (black bar) and G2/M (white bar) phases of the cell cycle. (B) Corresponding relative apoptotic activity normalized to untreated control relative cell number.

We examined the effect of PG-11047 on cell cycle distribution and apoptosis in order to understand the mechanism by which PG-11047-induced growth inhibition occurred. Six sensitive and three resistant cell lines were plated and treated as stated previously. These were labelled with BrdUrd at 48 and 72 h after PG-11047 treatment. The sensitive cell lines showed a greater reduction in fraction of cells incorporating BrdUrd than did the resistant cells. The cell cycle distributions of these cell lines treated with 0.3, 10 and 300 μM of PG-11047 for 72 h are shown in Figure 3A. There was a significant decrease in the fraction of cells in S-phase with increasing doses of PG-11047 in the cell lines that showed highest sensitivity; this effect was clearer at 72 h than at 48 h of exposure (data not shown). The three resistant cell lines, MDAMB436, MDAMB361 and SKBR3, showed only modest changes in the fractions of cells in S-phase. Apoptosis, measured with the Promega Caspase-Glo 3/7 assay, generally was induced at higher concentrations of PG-11047 than required to inhibit cell cycle traverse (Figure 3B) in sensitive cell lines. These data suggest that the growth inhibition induced by PG-11047 in these cell lines occurs more through cell cycle inhibition than by induction of apoptosis.

## Discussion

Polyamines are required for cellular viability and elevated levels are found in many tumour types, including breast cancer [2], making polyamine synthesis an attractive target for chemotherapy. PG-11047 is a second generation polyamine analogue specifically designed as a therapeutic agent [7]. While it is reported to effectively inhibit cell growth in lung, breast and colon cancer cell lines [7,12,24], only a limited number of cell lines had previously been studied. Since breast cancer is now known to be comprised of multiple genomic and transcriptional subsets [10,14] that progress and respond to therapy differently, we analysed quantitative responses to PG-11047 in a collection of 42 breast cancer and six non-malignant breast cell lines in order to identify biological and molecular features associated with response. Figure 1 shows that the basal subtype is most strongly inhibited by treatment with PG-11047, based on GI_50 _response profiles. Basal subtype breast cell lines mirror many molecular features of basal-like primary breast tumours [15,16] including low expression of oestrogen receptor and ERBB2 and high expression of keratin 5/6/14 and EGFR (Spellman *et al*., personal communication). This suggests that, PG-11047 may be preferentially effective against this more aggressive breast cancer subtype. This is consistent with observations made in a study of the polyamine analogue, *N*^1^, *N*^11^-diethylnorspermine (DENSPM) [25]. An analysis of BrdUrd incorporation and apoptosis induction in sensitive and resistant cell lines suggests that responses to micromolar concentrations of PG-11047 mainly involve reduced cell cycle traverse rather than induction of apoptosis. Holst *et al*. [13] also reported inhibition of growth in the breast cancer cell lines with PG-11047, although they observed a stronger apoptotic response. This discrepancy might be explained by differences in assay conditions, since they assessed apoptosis as an increase in the fraction of cells showing sub-G1 DNA content, while our study was based on analysis of apoptosis related caspases

The concentration of drug needed to inhibit growth measured as GI_50 _or TGI ranged over four orders of magnitude which meant that molecular features associated with response could be identified with confidence. The correlation of transcription profiles of the cell lines with their GI_50 _sensitivity identified 250 genes whose expression levels were associated with response to PG-11047. Network and pathway analyses of these genes are summarized in Figure 2 and Table 1. Increased interferon signaling was implicated with increased sensitivity to PG-11047 by both pathway and network analyses (Network 2). This is consistent with the observation that interferon inhibits the activity of ornithine decarboxylase (ODC) [26]. We speculate that cells with high interferon activity are preferentially sensitive to PG-11047 because interferon-induced down regulation of ODC reduces the endogenous pool of the polyamines with which PG-11047 must compete to affect its inhibitory functions. Other signalling pathways and networks implicated in Table 1 and Figure 2 have been reported to be differentially active in basal and luminal tumours and cell lines. For example, pathway activities associated with basal subtype tumours involve Ephrin receptor [14,27], BRCA1 [28] and integrins [29]. The strong basal subtype specificity of PG-11047 probably explains the associations with these pathway activities. Differential sensitivity of basal and luminal cells to PG-11047 also probably explains the structure of Network 1 in Figure 2, since this network included several genes with strong subtype specific expression [14].

A further analysis of the 250 genes associated with response to PG-11047 identified 13 genes whose levels were strongly and independently associated with response (Table 2). We propose that a clinical response predictor could be generated by assaying the levels of expression of these genes. To this end, we evaluated the applicability of the 13 gene set in stratifying breast tumour transcription datasets. We used the model to predict the sensitivities of the tumour samples in the Chin *et al*. [11] tumour panel (total = 118 samples). We found that there is a tendency toward separation between the predicted sensitivities of basal versus non-basal tumours (*P *= 0.08). The stratification improved if we restricted the analysis to high confidence predictions of the model (89/118 samples): *P *= 0.01. This analysis supports the utility of PG-11047 in the treatment of basal-subtype tumours.

Assessments of the contributions of these 13 response-associated-genes to breast cancer pathophysiology may provide insights into breast cancer responses to PG-11047 that were not directly tested in this study. Table 2 shows that increased sensitivity to PG-11047 is associated with lower expression of WASL, CST3, DEAF1 and ACSL3 and higher expression of GCLM, LAMA3, SSRP1, ACYP1, CYLD, PRPF18, AMFR, PPP1R2 and LOH11CR2A.

Several of these associations suggest that PG-11047 may act to inhibit aspects of migration/metastasis. For example, the protein encoded by CST3 is an antagonist of TGF-β signaling and has been shown to be significantly down regulated in many breast cancers [30]. Thus, PG-11047 may be preferentially effective in cells with active TGF-β signaling, a phenotype that has been associated with aggressive cancer behavior including increased migration/metastasis [31] Reduced expression of WASL and increased expression of AMFR also have been reported in breast cancer tissue [32,33] and associated with cellular migration [33-35]. Increased AMFR expression also is associated with increased phospho-AKT levels in primary human breast cancers [36]. Consistent with this, we observed that a higher protein level of phospho-AKT is a significant predictor for PG-11047 sensitive cells (Additional File 4). The implication of PG-11047 as an inhibitor of motility/metastasis is further supported by the fact that aspects of motility are known to be regulated by polyamines [37]. The associations of gains of the chromosomal regions near AMFR (16q21) and loss near CST3 (20p11.2) suggests that the associations of gene expression changes with motility/metastasis and that response to PG-11047 may be driven by genomic abnormalities.

Gene function assessments also suggest that PG-11047 may moderate aspects of epithelial to mesenchymal transition that are associated with aggressive clinical behaviour. For example, Carpenter *et al*. [38] have recently shown that laminin 5 may contribute to increased tumour aggressiveness resulting from epithelial to mesenchymal transition (EMT) in breast tumours. Increased expression of LAMA3, a subunit of laminin 5, is associated with response to PG-11047 suggesting the possibility that tumours responding to the drug also may experience reduced EMT. Among the other genes associated with response, GCLM and SSRP1 have been implicated in stress response [39,40], PRPF18 and SSRP1 are involved with transcription/translation [41,42], CYLD and PPP1R2 are involved with cell cycle regulation [43,44] and CYLD and LOH11CR2A have been reported as anti-oncogene/tumour suppressor genes [45,46].

## Conclusions

We assessed the quantitative responses of 48 breast cell lines to PG-11047 and demonstrated that there is a wide range of response to treatment. Our studies suggest that basal-subtype breast cancers are preferentially sensitive to the drug and that response at lower treatment concentrations is cytostatic rather than apoptotic. Analysis of molecular features associated with response suggests that PG-11047 may also reduce metastasis-related motility and suppress the EMT phenotype. We have generated a 13 gene set response signature to identify tumours expected to respond best to PG-11047 that may be useful in the selection of patients for further evaluation of PG-11047.

## Abbreviations

BESpm: bisethylspermine; BrdUrd: bromodeoxyuridine; CTG: CellTiter-Glo; GI: growth inhibition; EMT: epithelial to mesenchymal transition; IPA: Ingenuity Pathway Analysis; GI_50_: 50% GI; TGI: total growth inhibition; MCCV: Monte Carlo cross validation; ODC: ornithine decarboxylase; PBS: phosphate-buffered saline.

## Competing interests

LJM is an employee of Progen Pharmaceuticals. This work was supported in part by grant from Progen Pharmaceuticals.

## Authors' contributions

WK, RMN, JWG, LJM, ZH, NJW and HSF made substantial contributions to the conception and design of the study. WK, SZ and NB carried out the experiments and helped with the data acquisition. WK, SZ, DD, SB, PTS, WJB, LMH, AS and GVF helped with analysis and interpretation of the data. WK, DD, SZ, AJW, PTS, LJM and JWG were involved in drafting or critically revising the manuscript for important intellectual content. All authors gave their final approval of the version to be published.

## Pre-publication history

The pre-publication history for this paper can be accessed here:

http://www.biomedcentral.com/1741-7015/7/77/prepub

## Supplementary Material

Additional file 1 **Growth inhibition response (GI_50_, TGI) to PG-11047 and molecular features of breast cell lines**. GI_50 _and TGI for members of the breast cell lines calculated as describe in Methods are listed in decreasing GI_50 _sensitivity with subtype classification and ER/PR/HER2 status reported by Neve *et al*. [14] and Spellman *et al*. (personal communication). Click here for file

Additional file 2 **Statistically significant mRNA markers of response to PG-11047 (top 250 genes)**. Markers generated by correlation of growth inhibition (GI_50_) sensitivity with expression data of the cell lines reported by Neve *et al*[14]. Click here for file

Additional file 3 **Statistically significant genomic markers (BAC clones) of response to PG-11047**. Markers generated by correlation of growth inhibition (GI_50_) sensitivity with array CGH data of the cell lines reported by Neve *et al*. [14]. Bold text indicates clones located at or near four of the predictive markers. The chromosomal locations of these four predictive markers are also listed. Click here for file

Additional file 4 **Statistically significant protein markers of response to PG-11047**. Markers generated by correlation of growth inhibition (GI_50_) sensitivity with Western profile data of the cell lines reported by Neve *et al*. [14]. Click here for file
