# Supplementary material for: A systems analysis of the chemosensitivity of breast cancer cells to the polyamine analogue PG-11047
Source: BMC Med. 2009 Dec 14;7:77. doi: 10.1186/1741-7015-7-77 (PMC2803786; doi:10.1186/1741-7015-7-77)
Supplement: Additional file 1 — Growth inhibition response (GI50, TGI) to PG-11047 and molecular features of breast cell lines. GI50 and TGI for members of the breast cell lines calculated as describe in Methods are listed in decreasing GI50 sensitivity with subtype classification and ER/PR/HER2 status reported by Neve et al. [14] and Spellman et al. (personal communication). [file 1741-7015-7-77-S1.PDF]

Additional File 1. Growth inhibition response (GI_{50}, TGI) to PG-11047 and molecular features of breast cell lines.

		PG-11047		Expression Status		
**Cell Line**	**Subtype**	**GI50 (M)**	**TGI (M)**	**ER**	**PR**	**HER2**
HCC70	BaA	4.0E-07	5.3E-07	–	–	–
Hs578T	BaB	4.0E-07	5.0E-04	–	–	–
T47D	Lu	4.0E-07	2.0E-04	+	+	–
MDAMB468	BaA	5.0E-07	2.0E-03	–	–	–
HCC1806	BaA	6.0E-07	3.3E-04	–	–	–
HCC1937	BaA	8.0E-07	3.0E-04	–	–	–
S1	N	8.0E-07	3.0E-03			
ZR7530	Lu	8.0E-07	1.0E-03	+	–	+
BT549	BaB	1.0E-06	1.3E-03	–	–	–
HCC1428	Lu	1.0E-06	3.0E-04	+	+	–
HCC1954	BaA	1.0E-06	8.0E-06	–	–	+
HCC3153	BaA	1.3E-06	8.0E-06	–	–	–
184A1	N, BaA	2.0E-06	2.2E-04	–	–	–
600MPE	Lu	2.0E-06	2.0E-03	+	–	–
T4	BaB	2.0E-06	8.0E-04			
HCC1143	BaA	2.0E-06	3.2E-04	–	–	–
SUM149PT	BaA	2.0E-06	8.0E-04	–	–	–
HCC1419	Lu	7.0E-06	3.1E-05	+	–	+
SUM52PE	Lu	1.0E-05	3.0E-04	+	–	–
ZR751	Lu	1.3E-05	8.0E-04	+	–	–
HCC1500	BaA	2.0E-05	6.0E-04	–	–	–
MDAMB415	Lu	2.0E-05	1.0E-04	+	–	–
UACC812	Lu	2.1E-05	3.0E-04	+	–	+
MDAMB231	BaB	2.3E-05	4.0E-03	–	–	–
AU565	Lu	4.0E-05	>3E-5	–	–	+
HCC38	BaB	4.0E-05	3.0E-04	–	–	–
HCC2185	Lu	9.0E-05	5.0E-04	–	–	–
SUM229PE	BaB	9.0E-05	1.1E-03			
MCF7	Lu	9.0E-05	1.2E-03	+	–	–
MCF10F	N, BaA	1.0E-04	1.1E-03			
MCF12A	N, BaA	1.0E-04	>1E-4	–	–	–
MCF10A	N, BaA	2.0E-04	3.3E-03	–	–	–
MDAMB157	BaB	2.0E-04	1.0E-03	–	–	–
SUM159PT	BaB	2.0E-04	3.0E-03	–	–	–
ZR75B	Lu	2.0E-04	2.0E-03	+	–	–
HCC1569	BaA	2.2E-04	2.0E-03	–	–	+
184B5	N, BaA	3.0E-04	4.0E-04	–	–	–
SUM185PE	Lu	3.0E-04	>5E-4	–	–	–
CAMA1	Lu	3.5E-04	1.1E-03	+	+	+
BT474	Lu	4.0E-04	1.0E-03	+	+	+
MDAMB453	Lu	5.0E-04	>5e-4	–	–	–
MDAMB361	Lu	6.0E-04	2.0E-03	+	+	+

SUM1315MO2	BaB	7.0E-04	1.3E-03	–	–	–
BT483	Lu	9.0E-04	1.3E-03	+	+	–
MDAMB175VII	Lu	1.0E-03	2.0E-03	+	–	–
MDAMB436	BaB	1.0E-03	3.0E-03	–	–	–
LY2	Lu	1.3E-03	3.0E-03	+	–	–
SKBR3	Lu	5.0E-03	>5E-4	–	–	+

Subtype classification and ER/PR/HER2 status as reported by Neve *et al.* (13) and Spellman *et al.* (personal communication).

BaA: basal A, BaB: basal B, Lu: luminal, N: non-cancerous.
