# Supplementary material for: A systems analysis of the chemosensitivity of breast cancer cells to the polyamine analogue PG-11047
Source: BMC Med. 2009 Dec 14;7:77. doi: 10.1186/1741-7015-7-77 (PMC2803786; doi:10.1186/1741-7015-7-77)
Supplement: Additional file 2 — Statistically significant mRNA markers of response to PG-11047 (top 250 genes). Markers generated by correlation of growth inhibition (GI50) sensitivity with expression data of the cell lines reported by Neve et al[14]. [file 1741-7015-7-77-S2.PDF]

Additional File 2. Statistically significant mRNA markers of response to PG-11047 (top 250 genes).

Gene Symbol	p-value	q-value	Predicts Sensitivity (S) or Resistance (R)	Chromosomal Location	Description
MYCBP	3.5E-06	3.3E-03	S	chr1p33-p32.2	c-myc binding protein
RPL15	3.9E-06	3.3E-03	R	chr3p24.2	ribosomal protein L15
PPAP2C	7.3E-06	3.3E-03	S	chr19p13	phosphatidic acid phosphatase type 2C
LSR	1.4E-05	4.1E-03	S	chr19q13.12	lipolysis stimulated lipoprotein receptor
RABAC1	1.7E-05	4.4E-03	S	chr19q13.2	Rab acceptor 1 (prenylated)
NOVA1	2.5E-05	5.5E-03	R	chr14q	neuro-oncological ventral antigen 1
WASL	2.5E-05	5.5E-03	R	chr7q31.3	Wiskott-Aldrich syndrome-like
RARRES1	3.6E-05	6.4E-03	S	chr3q25.32-q25.33	retinoic acid receptor responder (tazarotene induced) 1
KRT23	4.3E-05	6.4E-03	S	chr17q21.2	keratin 23 (histone deacetylase inducible)
STAG2	4.4E-05	6.4E-03	S	chrXq25	stromal antigen 2
GCLM	4.7E-05	6.4E-03	S	chr1p22.1	glutamate-cysteine ligase, modifier subunit
UBE2Z	5.0E-05	6.4E-03	R	chr17q21.32	ubiquitin-conjugating enzyme E2Z (putative)
RBP1	5.8E-05	7.0E-03	S	chr3q23	retinol binding protein 1, cellular
CST3	6.3E-05	7.1E-03	R	chr20p11.21	cystatin C (amyloid angiopathy and cerebral hemorrhage)
ENPP1	6.4E-05	7.1E-03	R	chr6q22-q23	ectonucleotide pyrophosphatase/phosphodiesterase 1
VTCN1	6.5E-05	7.1E-03	S	chr1p13.1	V-set domain containing T cell activation inhibitor 1
LAMA3	6.7E-05	7.1E-03	S	chr18q11.2	laminin, alpha 3
RHOB	7.8E-05	7.6E-03	R	chr2p24	ras homolog gene family, member B
GSTZ1	8.3E-05	7.8E-03	R	chr14q24.3	glutathione transferase zeta 1 (maleylacetoacetate isomerase)
NUDT21	9.4E-05	8.2E-03	S	chr16q13	nudix (nucleoside diphosphate linked moiety X)-type motif 21
SFN	9.9E-05	8.2E-03	S	chr1p36.11	stratifin
MRPS35	1.0E-04	8.2E-03	R	chr12p11	mitochondrial ribosomal protein S35
CHI3L2	1.0E-04	8.2E-03	S	chr1p13.3	chitinase 3-like 2 /// chitinase 3-like 2
TMEM123	1.1E-04	8.2E-03	S	chr11q22.1	transmembrane protein 123
SSRP1	1.2E-04	8.4E-03	S	chr11q12	structure specific recognition protein 1
ACYP1	1.2E-04	8.4E-03	S	chr14q24.3	acylphosphatase 1, erythrocyte (common) type
UBXD2	1.2E-04	8.4E-03	S	chr2q21.3	UBX domain containing 2
CD59	1.3E-04	8.4E-03	S	chr11p13	CD59 molecule, complement regulatory protein
NMI	1.3E-04	8.4E-03	S	chr2p24.3-q21.3	N-myc (and STAT) interactor
C16orf80	1.7E-04	9.2E-03	S	chr16q21	chromosome 16 open reading frame 80
SRRM2	1.7E-04	9.2E-03	R	chr16p13.3	serine/arginine repetitive matrix 2
OSTF1	1.7E-04	9.2E-03	S	chr9q13-q21.2	osteoclast stimulating factor 1
MAX	1.8E-04	9.3E-03	R	chr14q23	MYC associated factor X
MGP	1.8E-04	9.3E-03	S	chr12p13.1-p12.3	matrix Gla protein
LAMB3	1.9E-04	9.5E-03	S	chr1q32	laminin, beta 3
C14orf112	2.0E-04	9.8E-03	R	chr14q24.2	Chromosome 14 open reading frame 112
PITX1	2.0E-04	9.8E-03	S	chr5q31	paired-like homeodomain transcription factor 1
AF146796	2.4E-04	1.1E-02	S	AF146796	---
RAP1A	2.5E-04	1.1E-02	S	chr1p13.3	RAP1A, member of RAS oncogene family
SRPK1	2.9E-04	1.1E-02	S	chr6p21.3-p21.2	SFRS protein kinase 1
CYLD	2.9E-04	1.1E-02	S	chr16q12.1	cylindromatosis (turban tumor syndrome)
PRPF18	3.0E-04	1.1E-02	S	chr10p13	PRP18 pre-mRNA processing factor 18 homolog (S. cerevisiae)
MAP4K4	3.0E-04	1.1E-02	S	chr2q11.2-q12	mitogen-activated protein kinase kinase kinase kinase 4
PELI1	3.0E-04	1.1E-02	S	chr2p13.3	pellino homolog 1 (Drosophila)
STK3	3.1E-04	1.1E-02	S	chr8q22.2	serine/threonine kinase 3 (STE20 homolog, yeast)
ABI1	3.1E-04	1.1E-02	S	chr10p11.2	abl-interactor 1
AMFR	3.1E-04	1.1E-02	S	chr16q21	autocrine motility factor receptor
ISGF3G	3.5E-04	1.2E-02	S	chr14q11.2	interferon-stimulated transcription factor 3, gamma 48kDa
HSPC152	3.6E-04	1.2E-02	R	chr11q13.1	hypothetical protein HSPC152
ZNF185	3.6E-04	1.2E-02	S	chrXq28	zinc finger protein 185 (LIM domain)

RBM35B	3.7E-04	1.2E-02	R	chr16q22.1	RNA binding motif protein 35B
ARHGAP29	3.8E-04	1.2E-02	S	chr1p22.1	Rho GTPase activating protein 29
RFC3	3.8E-04	1.2E-02	S	chr13q12.3-q13	replication factor C (activator 1) 3, 38kDa
AK2	4.1E-04	1.2E-02	S	chr1p34	adenylate kinase 2
C1orf80	4.2E-04	1.2E-02	S	chr1q41	chromosome 1 open reading frame 80
AI039469	4.4E-04	1.2E-02	R	AI039469	---
ALDH3A2	4.4E-04	1.2E-02	S	chr17p11.2	aldehyde dehydrogenase 3 family, member A2
XBP1	4.4E-04	1.2E-02	R	chr22q12.1|22q12	X-box binding protein 1
KRT17	4.5E-04	1.2E-02	S	chr17q12-q21	keratin 17
ARF4	4.5E-04	1.2E-02	S	chr3p21.2-p21.1	ADP-ribosylation factor 4
C5orf28	4.6E-04	1.2E-02	S	chr5p12	chromosome 5 open reading frame 28
DUSP4	4.6E-04	1.2E-02	R	chr8p12-p11	dual specificity phosphatase 4
KLF5	4.6E-04	1.2E-02	S	chr13q22.1	Kruppel-like factor 5 (intestinal)
SH2B3	4.8E-04	1.2E-02	R	chr12q24	SH2B adaptor protein 3
CEBPG	4.8E-04	1.2E-02	S	chr19q13.11	CCAAT/enhancer binding protein (C/EBP), gamma
GPSM2	5.0E-04	1.2E-02	S	chr1p13.3	G-protein signalling modulator 2 (AGS3-like, C. elegans)
LCN2	5.1E-04	1.2E-02	S	chr9q34	lipocalin 2 (oncogene 24p3)
LAMP3	5.1E-04	1.2E-02	S	chr3q26.3-q27	lysosomal-associated membrane protein 3
PTS	5.1E-04	1.2E-02	S	chr11q22.3-q23.3	6-pyruvoyltetrahydropterin synthase
RBM7	5.1E-04	1.2E-02	S	chr11q23.1-q23.2	RNA binding motif protein 7
PRO1843	5.1E-04	1.2E-02	R	chr12q13.13	hypothetical protein PRO1843
NRCAM	5.2E-04	1.2E-02	R	chr7q31.1-q31.2	neuronal cell adhesion molecule
BRD9	5.3E-04	1.2E-02	S	chr5p15.33	bromodomain containing 9
SERPINB5	5.3E-04	1.2E-02	S	chr18q21.3	serpin peptidase inhibitor, clade B (ovalbumin), member 5
RFP	5.3E-04	1.2E-02	S	chr6p22	ret finger protein
POLR3E	5.6E-04	1.3E-02	R	chr16p12.1	polymerase (RNA) III (DNA directed) polypeptide E (80kD)
LYN	5.7E-04	1.3E-02	S	chr8q13	v-yes-1 Yamaguchi sarcoma viral related oncogene homolog /// v-yes-1 Yamaguchi sarcoma viral related oncogene homolog
FOXA1	5.8E-04	1.3E-02	R	chr14q12-q13	forkhead box A1
ARFIP2	5.8E-04	1.3E-02	R	chr11p15	ADP-ribosylation factor interacting protein 2 (arfaptin 2)
EFHC1	6.1E-04	1.3E-02	R	chr6p12.3	EF-hand domain (C-terminal) containing 1
GNAI3	6.1E-04	1.3E-02	S	chr1p13	guanine nucleotide binding protein (G protein), alpha inhibiting activity polypeptide 3
USP25	6.2E-04	1.3E-02	S	chr21q11.2	ubiquitin specific peptidase 25
PFDN5	6.2E-04	1.3E-02	R	chr12q12	prefoldin subunit 5
TSPAN6 /// ZNF444	6.3E-04	1.3E-02	S	chrXq22 /// chr19q13.42	tetraspanin 6 /// zinc finger protein 444
WWTR1	6.4E-04	1.3E-02	S	chr3q23-q24	WW domain containing transcription regulator 1
KRT6B	6.6E-04	1.3E-02	S	chr12q12-q13	keratin 6B
CHMP7	6.7E-04	1.3E-02	R	chr8p21.3	CHMP family, member 7
PROM1	6.7E-04	1.3E-02	S	chr4p15.32	prominin 1
SPDEF	6.8E-04	1.3E-02	R	chr6p21.3	SAM pointed domain containing ets transcription factor
DCPS	6.8E-04	1.3E-02	S	chr11q24.2	decapping enzyme, scavenger
PTPRN2	6.8E-04	1.3E-02	R	chr7q36	protein tyrosine phosphatase, receptor type, N polypeptide 2
MPZL1	6.9E-04	1.3E-02	S	chr1q24.2	myelin protein zero-like 1
ASCC3	6.9E-04	1.3E-02	S	chr6q16.1-q16.3	activating signal cointegrator 1 complex subunit 3
JAG1	7.2E-04	1.4E-02	S	chr20p12.1-p11.23	jagged 1 (Alagille syndrome)
PLSCR1	7.3E-04	1.4E-02	S	chr3q23	phospholipid scramblase 1
CRNKL1	7.4E-04	1.4E-02	R	chr20p11.2	Crn, crooked neck-like 1 (Drosophila)
NEBL	7.5E-04	1.4E-02	R	chr10p12	nebulette
DEAF1	7.9E-04	1.4E-02	R	chr11p15.5	deformed epidermal autoregulatory factor 1 (Drosophila)
15-Sep	7.9E-04	1.4E-02	S	chr1p31	15 kDa selenoprotein
NUP93	7.9E-04	1.4E-02	S	chr16q13	nucleoporin 93kDa
EVI2A	8.0E-04	1.4E-02	R	chr17q11.2	ecotropic viral integration site 2A

MGC10433	8.0E-04	1.4E-02	S	chr19q13.12	hypothetical protein MGC10433
E2F3	8.2E-04	1.4E-02	S	chr6p22	E2F transcription factor 3
PLS1	8.2E-04	1.4E-02	S	chr3q23	plastin 1 (I isoform)
KAL1	8.2E-04	1.4E-02	R	chrXp22.32	Kallmann syndrome 1 sequence
KRT5	8.4E-04	1.4E-02	S	chr12q12-q13	keratin 5 (epidermolysis bullosa simplex, Dowling-Meara/Kobner/Weber-Cockayne types)
CROCC /// MGC12760 /// LOC645719	8.5E-04	1.4E-02	S	chr1pter-p36.11 /// chr1p36.13	ciliary rootlet coiled-coil, rootletin /// ciliary rootlet coiled-coil, rootletin /// hypothetical protein MGC12760 /// hypothetical protein MGC12760 /// similar to ciliary rootlet coiled-coil, rootletin /// similar to ciliary rootlet coiled-coil, rootletin
AKR1C2	8.8E-04	1.4E-02	S	chr10p15-p14	aldo-keto reductase family 1, member C2 (dihydrodiol dehydrogenase 2; bile acid binding protein; 3-alpha hydroxysteroid dehydrogenase, type III)
GMPS	9.0E-04	1.5E-02	S	chr3q24	guanine monphosphate synthetase
KRT14	9.2E-04	1.5E-02	S	chr17q12-q21	keratin 14 (epidermolysis bullosa simplex, Dowling-Meara, Koebner)
C14orf132	9.2E-04	1.5E-02	R	chr14q32.2	chromosome 14 open reading frame 132
BNIP3	9.5E-04	1.5E-02	R	chr10q26.3	BCL2/adenovirus E1B 19kDa interacting protein 3
STXBP3	9.6E-04	1.5E-02	S	chr1p13.3	syntaxin binding protein 3
LPL	9.6E-04	1.5E-02	R	chr8p22	lipoprotein lipase
C6orf75	9.9E-04	1.5E-02	S	chr6q11.1-q22.33	chromosome 6 open reading frame 75
SAA1	1.0E-03	1.5E-02	S	chr11p15.1	serum amyloid A1
MSH2	1.0E-03	1.5E-02	S	chr2p22-p21	mutS homolog 2, colon cancer, nonpolyposis type 1 (E. coli)
CTSH	1.0E-03	1.5E-02	S	chr15q24-q25	cathepsin H
PPP2R5A	1.1E-03	1.6E-02	S	chr1q32.2-q32.3	protein phosphatase 2, regulatory subunit B (B56), alpha isoform
CDH3	1.1E-03	1.6E-02	S	chr16q22.1	cadherin 3, type 1, P-cadherin (placental)
ZNF706	1.1E-03	1.6E-02	S	chr8q22.3	zinc finger protein 706
SPATA20	1.1E-03	1.6E-02	R	chr17q21.33	spermatogenesis associated 20
C1orf116 /// LOC653098	1.1E-03	1.6E-02	S	chr1q32.1	chromosome 1 open reading frame 116 /// similar to chromosome 1 open reading frame 116
DNAJA1	1.1E-03	1.6E-02	R	chr9p13-p12	DnaJ (Hsp40) homolog, subfamily A, member 1
GTPBP8	1.1E-03	1.6E-02	S	chr3q13.2	GTP-binding protein 8 (putative)
HNRPUL1	1.1E-03	1.6E-02	S	chr19q13.2	heterogeneous nuclear ribonucleoprotein U-like 1
IFI27	1.1E-03	1.6E-02	S	chr14q32	interferon, alpha-inducible protein 27
LOC653468 /// LOC653471	1.2E-03	1.6E-02	R	chr10q22.2	hypothetical protein LOC653468 /// similar to Ribosome biogenesis protein BMS1 homolog
NFASC	1.2E-03	1.6E-02	S	chr1q32.1	neurofascin homolog (chicken)
TMEM45A	1.2E-03	1.6E-02	S	chr3q12.2	transmembrane protein 45A
FOXJ3	1.2E-03	1.6E-02	S	chr1pter-q31.3	forkhead box J3
YWHAH	1.2E-03	1.6E-02	S	chr22q12.3	tyrosine 3-monooxygenase/tryptophan 5-monooxygenase activation protein, eta polypeptide
CTDSP1	1.2E-03	1.7E-02	R	chr2q35	CTD (carboxy-terminal domain, RNA polymerase II, polypeptide A) small phosphatase 1
UBN1	1.2E-03	1.7E-02	R	chr16p13.3	ubinuclein 1
HMGN3	1.3E-03	1.7E-02	S	chr6q14.1	high mobility group nucleosomal binding domain 3
BID	1.3E-03	1.7E-02	S	chr22q11.1	BH3 interacting domain death agonist /// BH3 interacting domain death agonist
DR1	1.3E-03	1.7E-02	S	chr1p22.1	down-regulator of transcription 1, TBP-binding (negative cofactor 2)
IFI30	1.3E-03	1.7E-02	S	chr19p13.1	interferon, gamma-inducible protein 30
SLC9A3R1	1.3E-03	1.7E-02	R	chr17q25.1	solute carrier family 9 (sodium/hydrogen exchanger), member 3 regulator 1
C20orf7 /// TMEM14B	1.3E-03	1.7E-02	S	chr20p12.1 /// chr6p25.1-p23	chromosome 20 open reading frame 7 /// chromosome 20 open reading frame 7 /// transmembrane protein 14B /// transmembrane protein 14B
NINJ1	1.3E-03	1.7E-02	R	chr9q22	ninjurin 1
TMEM30A	1.3E-03	1.7E-02	S	chr6q14.1	transmembrane protein 30A
EIF4B	1.3E-03	1.7E-02	R	chr12q13.13	eukaryotic translation initiation factor 4B
RPS6KB1	1.4E-03	1.7E-02	R	chr17q23.1	ribosomal protein S6 kinase, 70kDa, polypeptide 1

CSPG2	1.4E-03	1.7E-02	R	chr5q14.3	chondroitin sulfate proteoglycan 2 (versican)
IFIH1	1.5E-03	1.7E-02	S	chr2p24.3-q24.3	interferon induced with helicase C domain 1
ECHDC2	1.5E-03	1.7E-02	S	chr1p32.3	enoyl Coenzyme A hydratase domain containing 2
PDZK1IP1	1.5E-03	1.7E-02	S	chr1p33	PDZK1 interacting protein 1
FBN2	1.5E-03	1.7E-02	R	chr5q23-q31	fibrillin 2 (congenital contractural arachnodactyly)
TNFAIP8	1.5E-03	1.7E-02	S	chr5q23.1	tumor necrosis factor, alpha-induced protein 8
SRP19	1.5E-03	1.7E-02	S	chr5q21-q22	signal recognition particle 19kDa
EPPK1	1.5E-03	1.7E-02	S	chr8q24.3	epiplakin 1 /// epiplakin 1
HNRPA0	1.5E-03	1.7E-02	R	chr5q31	heterogeneous nuclear ribonucleoprotein A0
RPA1	1.5E-03	1.7E-02	S	chr17p13.3	replication protein A1, 70kDa
NDUFA4	1.5E-03	1.7E-02	R	chr7p21.3	NADH dehydrogenase (ubiquinone) 1 alpha subcomplex, 4, 9kDa
SIRT1	1.5E-03	1.7E-02	R	chr10q21.3	sirtuin (silent mating type information regulation 2 homolog) 1 (S. cerevisiae)
PPP1R2	1.5E-03	1.7E-02	S	chr3q29	protein phosphatase 1, regulatory (inhibitor) subunit 2
TAOK3	1.5E-03	1.7E-02	S	chr12q	TAO kinase 3
ANXA3	1.5E-03	1.7E-02	S	chr4q13-q22	annexin A3
FDX1	1.6E-03	1.7E-02	S	chr11q22	ferredoxin 1
KIAA0100	1.6E-03	1.7E-02	R	chr17q11.2	KIAA0100
NOC2L	1.6E-03	1.7E-02	S	chr1p36.33	nucleolar complex associated 2 homolog (S. cerevisiae)
IFNGR1	1.6E-03	1.8E-02	S	chr6q23-q24	interferon gamma receptor 1
S100A2	1.6E-03	1.8E-02	S	chr1q21	S100 calcium binding protein A2
LOH11CR2A	1.7E-03	1.8E-02	S	chr11q23	loss of heterozygosity, 11, chromosomal region 2, gene A
TFG	1.7E-03	1.8E-02	S	chr3q12.2	TRK-fused gene
CPNE3	1.7E-03	1.8E-02	S	chr8q21.3	copine III
BIRC1 /// LOC648984 /// LOC653371	1.7E-03	1.8E-02	R	chr5q13.1 /// chr5q13.2	baculoviral IAP repeat-containing 1 /// similar to Baculoviral IAP repeat-containing protein 1 (Neuronal apoptosis inhibitory protein) /// similar to Baculoviral IAP repeat-containing protein 1 (Neuronal apoptosis inhibitory protein)
FANCL	1.7E-03	1.8E-02	S	chr2p16.1	Fanconi anemia, complementation group L
EIF3S10	1.7E-03	1.8E-02	R	chr10q26	eukaryotic translation initiation factor 3, subunit 10 theta, 150/170kDa
ACSL3	1.8E-03	1.8E-02	R	chr2q34-q35	acyl-CoA synthetase long-chain family member 3
C14orf78	1.8E-03	1.8E-02	S	chr14q32.33	chromosome 14 open reading frame 78
SDF4	1.8E-03	1.8E-02	S	chr1p36.33	stromal cell derived factor 4
CRYAB	1.8E-03	1.8E-02	S	chr11q22.3-q23.1	crystallin, alpha B
LSM3	1.8E-03	1.8E-02	R	chr3p25.1	LSM3 homolog, U6 small nuclear RNA associated (S. cerevisiae)
COTL1	1.9E-03	1.8E-02	S	chr16q24.1	coactosin-like 1 (Dictyostelium)
CENPA	1.9E-03	1.8E-02	S	chr2p24-p21	centromere protein A
SERHL2	1.9E-03	1.8E-02	R	chr22q13	serine hydrolase-like 2
STX6	1.9E-03	1.9E-02	S	chr1q25.3	syntaxin 6
CADPS2	1.9E-03	1.9E-02	R	chr7q31.3	Ca2+-dependent activator protein for secretion 2
PAK1	1.9E-03	1.9E-02	S	chr11q13-q14	p21/Cdc42/Rac1-activated kinase 1 (STE20 homolog, yeast)
USP48	1.9E-03	1.9E-02	R	chr1p36.12	ubiquitin specific peptidase 48
CES2	1.9E-03	1.9E-02	S	chr16q22.1	carboxylesterase 2 (intestine, liver)
RPS27L	2.0E-03	1.9E-02	R	chr15q22.2	ribosomal protein S27-like
CASP1	2.0E-03	1.9E-02	S	chr11q23	caspase 1, apoptosis-related cysteine peptidase (interleukin 1, beta, convertase)
CACNA2D2	2.0E-03	1.9E-02	R	chr3p21.3	calcium channel, voltage-dependent, alpha 2/delta subunit 2
CUL2	2.0E-03	1.9E-02	S	chr10p11.21	cullin 2
ENPP2	2.0E-03	1.9E-02	R	chr8q24.1	ectonucleotide pyrophosphatase/phosphodiesterase 2 (autotaxin)
THUMPD1	2.0E-03	1.9E-02	R	chr16p12.2	THUMP domain containing 1
C1orf27	2.0E-03	1.9E-02	R	chr1q25	chromosome 1 open reading frame 27
CPT1A	2.1E-03	1.9E-02	R	chr11q13.1-q13.2	carnitine palmitoyltransferase 1A (liver)
UCRC	2.1E-03	1.9E-02	R	chr22cen-q12.3	ubiquinol-cytochrome c reductase complex (7.2 kD)
CRBN	2.1E-03	1.9E-02	R	chr3p26.3	cereblon
FAM111A	2.1E-03	1.9E-02	S	chr11q12.1	family with sequence similarity 111, member A

MBNL1	2.2E-03	2.0E-02	S	chr3q25	muscleblind-like (Drosophila)
TOM1L1	2.2E-03	2.0E-02	R	chr17q23.2	target of myb1-like 1 (chicken)
SUMO3	2.2E-03	2.0E-02	S	chr21q22.3	SMT3 suppressor of mif two 3 homolog 3 (S. cerevisiae)
IRF3	2.2E-03	2.0E-02	S	chr19q13.3-q13.4	interferon regulatory factor 3
PTBP1	2.2E-03	2.0E-02	S	chr19p13.3	polypyrimidine tract binding protein 1
KRT15	2.2E-03	2.0E-02	S	chr17q21.2	keratin 15
SLPI	2.2E-03	2.0E-02	S	chr20q12	secretory leukocyte peptidase inhibitor
SCPEP1	2.3E-03	2.0E-02	S	chr17q22	serine carboxypeptidase 1
VPS16	2.3E-03	2.0E-02	R	chr20p13-p12	vacuolar protein sorting 16 (yeast)
NUP155	2.3E-03	2.0E-02	S	chr5p13.1	nucleoporin 155kDa
DERL2	2.3E-03	2.0E-02	S	chr17p	Der1-like domain family, member 2
CSPP1	2.4E-03	2.0E-02	R	chr8q13.2	centrosome and spindle pole associated protein 1
HSPA4	2.4E-03	2.0E-02	S	chr5q31.1-q31.2	heat shock 70kDa protein 4
NUP153	2.4E-03	2.0E-02	S	chr6p22.3	nucleoporin 153kDa
COPB2	2.4E-03	2.0E-02	R	chr3q23	Coatomer protein complex, subunit beta 2 (beta prime)
CAV2	2.4E-03	2.0E-02	S	chr7q31.1	caveolin 2
FBXL7	2.4E-03	2.0E-02	R	chr5p15.1	F-box and leucine-rich repeat protein 7
NNMT	2.4E-03	2.0E-02	S	chr11q23.1	nicotinamide N-methyltransferase
R3HDM1	2.5E-03	2.1E-02	S	chr2q21.3	R3H domain containing 1
HTATSF1	2.5E-03	2.1E-02	S	chrXq26.1-q27.2	HIV-1 Tat specific factor 1
AHR	2.5E-03	2.1E-02	S	chr7p15	aryl hydrocarbon receptor
RANBP1	2.6E-03	2.1E-02	S	chr22q11.21	RAN binding protein 1
RPL36AL	2.6E-03	2.1E-02	R	chr14q21	ribosomal protein L36a-like
HLA-F	2.6E-03	2.1E-02	S	chr6p21.3	major histocompatibility complex, class I, F
IER5	2.6E-03	2.1E-02	S	chr1q25.3	immediate early response 5
HARSL	2.6E-03	2.1E-02	R	chr5q31.3	histidyl-tRNA synthetase-like
PCNA	2.7E-03	2.1E-02	S	chr20pter-p12	proliferating cell nuclear antigen
ABR	2.7E-03	2.1E-02	R	chr17p13.3	active BCR-related gene
TCF4	2.7E-03	2.1E-02	R	chr18q21.1	transcription factor 4
UBE2M	2.7E-03	2.1E-02	S	chr19q13.43	ubiquitin-conjugating enzyme E2M (UBC12 homolog, yeast)
RDBP	2.7E-03	2.1E-02	S	chr6p21.3	RD RNA binding protein
OAS3	2.7E-03	2.1E-02	S	chr12q24.2	2'-5'-oligoadenylate synthetase 3, 100kDa
TAP1	2.7E-03	2.1E-02	S	chr6p21.3	transporter 1, ATP-binding cassette, sub-family B (MDR/TAP)
GOLGA3	2.8E-03	2.2E-02	R	chr12q24.33	golgi autoantigen, golgin subfamily a, 3
MBNL2	2.8E-03	2.2E-02	S	chr13q32.1	muscleblind-like 2 (Drosophila)
MAPK1	2.9E-03	2.2E-02	S	chr22q11.2|22q11.21	mitogen-activated protein kinase 1
GBP1	2.9E-03	2.2E-02	S	chr1p22.2	guanylate binding protein 1, interferon-inducible, 67kDa /// guanylate binding protein 1, interferon-inducible, 67kDa
NDRG2	2.9E-03	2.2E-02	S	chr14q11.2	NDRG family member 2
HNRPDL	2.9E-03	2.2E-02	R	chr4q13-q21	heterogeneous nuclear ribonucleoprotein D-like
FABP5 /// LOC653327	3.0E-03	2.2E-02	S	chr8q21.13 /// chr11q12.1	fatty acid binding protein 5 (psoriasis-associated) /// similar to Fatty acid-binding protein, epidermal (E-FABP) (Psoriasis-associated fatty acid-binding protein homolog) (PA-FABP)
IRF1	3.0E-03	2.2E-02	S	chr5q31.1	interferon regulatory factor 1
WDR1	3.0E-03	2.2E-02	S	chr4p16.1	WD repeat domain 1
TM2D3	3.1E-03	2.2E-02	S	chr15q26.3	TM2 domain containing 3 /// TM2 domain containing 3
RP6-213H19.1	3.1E-03	2.2E-02	S	chrXq26.2	serine/threonine protein kinase MST4
SETD5	3.1E-03	2.2E-02	R	chr3p25.3	SET domain containing 5
CUEDC1	3.1E-03	2.3E-02	R	chr17q23.2	CUE domain containing 1
NUP37	3.2E-03	2.3E-02	S	chr12q23.2	nucleoporin 37kDa
HDAC2	3.2E-03	2.3E-02	S	chr6q21	histone deacetylase 2
ID4	3.2E-03	2.3E-02	S	chr6p22-p21	inhibitor of DNA binding 4, dominant negative helix-loop-helix protein
CXCR4	3.2E-03	2.3E-02	R	chr2q21	chemokine (C-X-C motif) receptor 4
STK38	3.2E-03	2.3E-02	S	chr6p21	serine/threonine kinase 38
APIP	3.3E-03	2.3E-02	S	chr11p13	APAF1 interacting protein

ASXL2	3.3E-03	2.3E-02	S	chr2p24.1	additional sex combs like 2 (Drosophila)
VAMP3	3.3E-03	2.3E-02	S	chr1p36.23	vesicle-associated membrane protein 3 (cellubrevin)
MATN2	3.3E-03	2.3E-02	S	chr8q22	matrilin 2
