# Supplementary material for: A systems analysis of the chemosensitivity of breast cancer cells to the polyamine analogue PG-11047
Source: BMC Med. 2009 Dec 14;7:77. doi: 10.1186/1741-7015-7-77 (PMC2803786; doi:10.1186/1741-7015-7-77)
Supplement: Additional file 3 — Statistically significant genomic markers (BAC clones) of response to PG-11047. Markers generated by correlation of growth inhibition (GI50) sensitivity with array CGH data of the cell lines reported by Neve et al. [14]. Bold text indicates clones located at or near four of the predictive markers. The chromosomal locations of these four predictive markers are also listed. [file 1741-7015-7-77-S3.PDF]

Additional File 3. Statistically significant genomic markers (BAC clones) of response to PG-11047.

Probe_id	p-value	q-value	Predicts ensitivity (S) or Resistance (R)	ChromosomeDistance (kbp)
RP11-210C7	2.03E-05	1.35E-02	S	19_48859.097
RP11-127K23	1.64E-04	2.28E-02	S	11_33268.592
LLNLF-119G3	3.13E-04	2.43E-02	R	19_3714.309
RP11-246O4	3.17E-04	2.43E-02	S	1_82663.328
RP11-148K15	3.28E-04	2.43E-02	R	1_201749.09
RP11-5J6	3.56E-04	2.43E-02	R	12_66705.892
RP11-862G10	3.75E-04	2.43E-02	S	11_120498.664
RP11-102C11	4.47E-04	2.58E-02	S	11_33538.818
RP11-260A10	4.64E-04	2.58E-02	R	1_214205.456
CTB-51J22	4.88E-04	2.58E-02	R	7_72822.38
RP11-206O6	5.88E-04	2.81E-02	S	19_33315.121
RP11-92J4	6.02E-04	2.81E-02	S	19_41164.177
RP11-267L1	6.09E-04	2.81E-02	S	11_97425.705
**RP11-61I15**	**6.82E-04**	**2.86E-02**	**S**	**16_55043.099**
RP11-10D18	7.96E-04	2.86E-02	R	20_51181.908
CTD-2007G21	1.04E-03	3.24E-02	R	7_93098.318
RP11-172G5	1.13E-03	3.24E-02	S	3_174406.67
RP11-61L22	1.15E-03	3.24E-02	S	22_43995.391
RP11-51J14	1.30E-03	3.24E-02	S	11_33309.957
RP11-246N19	1.54E-03	3.49E-02	S	1_63824.624
RP11-643C12	1.58E-03	3.49E-02	S	14_52853.763
RP11-271P14	1.76E-03	3.54E-02	S	11_121843.593
RP11-219O3	1.80E-03	3.54E-02	S	11_36373.104
LBL#1B7	1.81E-03	3.54E-02	S	20_614.133
RP11-227M19	1.82E-03	3.54E-02	S	5_3994.602
RP1-97B16	1.89E-03	3.54E-02	S	3_180272.87
RP11-283O4	1.91E-03	3.54E-02	S	11_33694.664
RP11-109J21	2.08E-03	3.73E-02	S	16_56004.898
RP11-114G6	2.17E-03	3.83E-02	S	11_40341.432
RP11-148D23	2.29E-03	3.84E-02	S	3_174686.842
RP11-215F2	2.42E-03	3.85E-02	S	11_31327.585
RP11-3D14	2.43E-03	3.85E-02	S	1_100485.231
CTD-2015N4	2.49E-03	3.89E-02	R	7_86105.763
RP11-66P13	2.61E-03	3.93E-02	S	10_26492.582
RP11-77O20	2.64E-03	3.93E-02	S	10_5586.356
RP11-72A10	2.81E-03	3.94E-02	S	11_36741.041
RP11-193J5	3.01E-03	4.17E-02	S	1_163240.621
RP11-150D18	3.04E-03	4.17E-02	S	11_41866.014
**RP11-135H8**	**3.19E-03**	**4.21E-02**	**S**	**11_56376.158**
**RP11-335F8**	**3.41E-03**	**4.22E-02**	**S**	**11_122198.129**
RP11-262J9	3.73E-03	4.22E-02	S	11_111244.499
RP11-246J15	3.80E-03	4.22E-02	S	1_199369.319
RP11-248G21	3.81E-03	4.22E-02	S	11_121238.097
RP11-66F24	3.83E-03	4.22E-02	S	10_22666.297
RP11-241D13	3.91E-03	4.22E-02	S	11_107572.975
RP11-164B14	3.94E-03	4.22E-02	S	11_121835.436
RP11-823O21	4.11E-03	4.22E-02	S	11_116450.955
RP11-102N4	4.13E-03	4.22E-02	S	11_36673.613

RP11-238I2	4.19E-03	4.22E-02	R	20_13575.058
RP11-82M24	4.19E-03	4.22E-02	S	5_3449.234
RP11-131C4	4.20E-03	4.22E-02	R	17_50764.493
RP11-24M13	4.31E-03	4.22E-02	R	16_8153.667
RP11-29N3	4.39E-03	4.22E-02	S	5_11435.333
RP11-47D7	4.49E-03	4.22E-02	R	11_11610.582
RP11-262M8	4.67E-03	4.22E-02	R	14_50687.245
CTD-2177J24	4.72E-03	4.22E-02	R	17_54012.047
RP5-1073F15	4.86E-03	4.22E-02	R	17_58373.574
RP11-69N2	4.88E-03	4.22E-02	S	10_340.36
RP11-147L8	4.91E-03	4.22E-02	S	11_30639.833
CTB-136O14	4.91E-03	4.22E-02	R	12_67488.237
RP11-19H11	4.93E-03	4.22E-02	S	19_48973.202
RP11-103L11	4.95E-03	4.22E-02	S	5_3816.063
RP11-96N14	5.10E-03	4.22E-02	S	11_121209.407
RP11-113M18	5.13E-03	4.22E-02	S	11_29797.991
RP11-32C15	5.30E-03	4.22E-02	R	7_95227.094
RP11-246J1	5.34E-03	4.22E-02	S	11_30504.859
RP11-62G18	5.41E-03	4.22E-02	R	11_19596.326
LLNLR-243D10	5.52E-03	4.22E-02	S	19_10105.175
RP11-124D1	5.56E-03	4.22E-02	R	20_47923.743
**RP11-2I4**	**5.62E-03**	**4.22E-02**	**S**	**16_55603.341**
RP11-70B16	5.88E-03	4.33E-02	S	10_34315.481
RP11-27H17	5.94E-03	4.33E-02	S	11_133589.317
RP11-181I11	5.95E-03	4.33E-02	S	10_30577.3
RP11-90F13	5.98E-03	4.33E-02	S	11_34775.916
LLNLR-272E5	6.07E-03	4.33E-02	S	19_10365.89
RP11-6H14	6.08E-03	4.33E-02	S	10_2197.185
RP11-775A2	6.33E-03	4.40E-02	S	11_119745.597
RP11-18J23	6.34E-03	4.40E-02	S	19_48075.706
RP11-37L21	6.50E-03	4.48E-02	R	10_102391.122
RP11-46I12	6.68E-03	4.55E-02	S	19_34301.924
LLNLR-262G12	6.89E-03	4.60E-02	S	19_4695.708
RP11-75H24	7.01E-03	4.60E-02	S	11_58499.504
RP11-339F13	7.12E-03	4.60E-02	S	7_54828.553
CTB-82N15	7.18E-03	4.60E-02	R	12_67488.237
GS-126B28	7.40E-03	4.60E-02	S	10_122902.442
RP11-163F8	7.42E-03	4.60E-02	S	9_17487.085
RP11-5N6	7.61E-03	4.60E-02	S	11_112963.87
GS1-207P11	7.64E-03	4.60E-02	R	7_85864.157
RP11-44F14	7.66E-03	4.60E-02	S	16_53247.001
RP11-163E9	7.71E-03	4.60E-02	R	7_101565.207
RP11-380G5	7.84E-03	4.60E-02	S	10_89271.554
RP11-112M22	7.85E-03	4.60E-02	S	11_127679.795
RP11-132M21	7.86E-03	4.60E-02	S	16_57967.271
RP5-1081P3	7.91E-03	4.60E-02	R	17_57549.987
RP11-772G14	8.07E-03	4.60E-02	S	11_118838.358
RP11-93E4	8.08E-03	4.60E-02	S	11_122168.351
RP11-41F17	8.26E-03	4.61E-02	R	10_71739.93
RP11-19L14	8.29E-03	4.61E-02	S	10_15557.234
**RP11-77C2**	**8.50E-03**	**4.61E-02**	**R**	**20_23093.12**
RP11-34I5	8.58E-03	4.61E-02	S	10_21377.644

CTD-2115C17	8.59E-03	4.61E-02	S	11_32373.794
RP11-27F2	8.60E-03	4.61E-02	S	10_133055.266
RP11-812L16	8.64E-03	4.61E-02	S	11_121336.737
RP11-50C21	8.73E-03	4.62E-02	S	9_10431.717
RP11-137E8	8.78E-03	4.62E-02	R	7_73356.816

Correlation of GI_{50} sensitivity with aCGH data of the cell lines reported by Neve *et al*. (13).

BOLD indicate clones located at or near the predictive markers.

The chromosomal distance of the four predictive markers are as follows:

Gene Symbol	Prediction	Chr location	Chromosome distance (kbp)
SSRP1	S	chr11q12	chr11:56850.034
LOH11CR2A	S	chr11q23	chr11:123491.343
AMFR	S	chr16q21	chr16:54952.893
CST3	R	chr20p11.21	chr20:23562.296
