# Supplementary material for: A systems analysis of the chemosensitivity of breast cancer cells to the polyamine analogue PG-11047
Source: BMC Med. 2009 Dec 14;7:77. doi: 10.1186/1741-7015-7-77 (PMC2803786; doi:10.1186/1741-7015-7-77)
Supplement: Additional file 4 — Statistically significant protein markers of response to PG-11047. Markers generated by correlation of growth inhibition (GI50) sensitivity with Western profile data of the cell lines reported by Neve et al. [14]. [file 1741-7015-7-77-S4.PDF]

Additional File 4. Statistically significant protein markers of response to PG-11047.

Probe id	p-value	q-value	Predicts Sensitivity (S) or Resistance (R)	Gene Symbol	Chromosome Location
AKT1-P	7.64E-04	3.36E-02	S	AKT1	14q32.32
RARA	3.00E-03	5.66E-02	S	RARA	17q21
MMP7	3.86E-03	5.66E-02	S	MMP7	11q21-q22
SKP2	7.54E-03	7.70E-02	R	SKP2	5p13
AKT1	1.31E-02	7.70E-02	R	AKT1	14q32.33
SFN	1.40E-02	7.70E-02	S	SFN	1p36.11
CBL	1.62E-02	7.70E-02	S	CBL	11q23.3
CCND1	1.77E-02	7.70E-02	R	CCND1	11q13
CK5/6	1.89E-02	7.70E-02	S	CK5/6	12q13.13
MAPK3	1.89E-02	7.70E-02	S	MAPK3	16p11.2
MEK-P	1.92E-02	7.70E-02	R	MAP2K1 (MEK1)	15q22.1-q22.33
CK18	3.37E-02	1.20E-01	R	CK18	12q13.13
IGF1R	3.56E-02	1.20E-01	R	IGF1R	15q26.3
CDH1	3.86E-02	1.21E-01	S	CDH1	16q22.1
CAV2	5.68E-02	1.63E-01	S	CAV2	7q31.2
